# Creatine Supplementation Improves Phosphagen Energy Pathway During Supramaximal Effort, but Does Not Improve Anaerobic Capacity or Performance

**DOI:** 10.3389/fphys.2019.00352

**Published:** 2019-04-10

**Authors:** Rodrigo de Araujo Bonetti de Poli, Luan Henrique Roncada, Elvis de Souza Malta, Guilherme Giannini Artioli, Rômulo Bertuzzi, Alessandro Moura Zagatto

**Affiliations:** ^1^Laboratory of Physiology and Sport Performance (LAFIDE), São Paulo State University (UNESP), Bauru, Brazil; ^2^Post-Graduate Program in Movement Sciences, São Paulo State University (UNESP), Bauru, Brazil; ^3^Department of Physical Education, School of Science, São Paulo State University (UNESP), Bauru, Brazil; ^4^Applied Physiology and Nutrition Research Group, University of São Paulo (USP), São Paulo, Brazil; ^5^School of Physical Education and Sport, University of São Paulo (USP), São Paulo, Brazil

**Keywords:** creatine, anaerobic capacity, energy contribution, high-intensity effort, performance

## Abstract

This study aimed to investigate the effects of short-duration creatine monohydrate supplementation on anaerobic capacity (AC), anaerobic energy pathways, and time-to-exhaustion during high-intensity running. Fourteen healthy men underwent a graded exercise test (GXT) followed by a O_2max_ confirmation test, 5 submaximal efforts, and 4 supramaximal running bouts at 115% of $\dot{V}$O_2max_ intensity (the first two supramaximal sessions were applied as familiarization trials) to measure the AC using two procedures; the maximum accumulated oxygen deficit (MAOD) and non-oxidative pathways energetics sum (AC_[La-]+EPOCfast_). The investigation was conducted in a single-blind and placebo-controlled manner, with participants performing the efforts first after being supplemented with a placebo (dextrose 20 g⋅day^-1^ for 5 days), and then, after a 7 day “placebo” washout period, they started the same procedure under creatine supplementation (20 g⋅day^-1^ for 5 days. This order was chosen due to the prolonged washout of creatine. MAOD was not different between placebo (3.35 ± 0.65 L) and creatine conditions (3.39 ± 0.79 L; P = 0.58) and presented a negligible effect [effect size (ES) = 0.08], similar to, AC_[La-]+EPOCfast_ (placebo condition (3.66 ± 0.79 Land under creatine ingestion 3.82 ± 0.85 L; P = 0.07) presenting a small effect (ES = 0.20). The energetics from the phosphagen pathway increased significantly after creatine supplementation (1.66 ± 0.40 L) compared to the placebo condition (1.55 ± 0.42 L; P = 0.03). However, the glycolytic and oxidative pathways were not different between conditions. Furthermore, time to exhaustion did not differ between placebo (160.79 ± 37.76 s) and creatine conditions (163.64 ± 38.72; P = 0.49). Therefore, we can conclude that creatine supplementation improves the phosphagen energy contribution, but with no statistical effect on AC or time to exhaustion in supramaximal running.

## Introduction

Creatine (α-methyl guanidine-acetic acid) is a nitrogen amine which can be obtained in diet (e.g., red and fish meat) and endogenously synthesized by the liver, kidneys, and pancreas (Hall and Trojian, 2013) and it is predominantly stored in skeletal muscle (≈95%) in both its free and phosphorylated forms (i.e., phosphorylcreatine) (Persky and Brazeau, 2001). One of the major roles of creatine is to act as a non-mitochondrial energy buffer, rapidly transferring energy through a reversible reaction catalyzed by the creatine kinase enzyme (Gualano et al., 2010).

Short-term creatine monohydrate supplementation has been widely used to improve performance in high-intensity and short-term efforts in cycling (Jacobs et al., 1997; Volek and Rawson, 2004; Hall and Trojian, 2013). Its effects have been mainly associated with increased intramuscular stores of creatine (∼+20%) (Harris et al., 1992) and increased phosphorylcreatine resynthesis rate (Greenhaff et al., 1994). Since creatine supplementation can significantly increase phosphorylcreatine intramuscular stores, it has been shown to improve the energy supply from the phosphagen systems (ePCr) (Yquel et al., 2002; Bemben and Lamont, 2005), thereby increasing the maximum capacity to resynthesize adenosine triphosphate (ATP) by non-oxidative pathways [i.e., anaerobic capacity (AC)] during high-intensity exercise. These changes could ultimately lead to improved performance in this type of exercise (Doherty et al., 2000).

Jacobs et al. (1997) verified the effects of short-duration creatine supplementation (20 g⋅day^-1^ for 5 days) on AC, measured using the maximal accumulated oxygen deficit (MAOD), and in supramaximal effort performance at 125% of the maximal oxygen uptake ($\dot{V}$O_2max_ ). These authors reported an increase of ≈9% in MAOD and ≈8% in time-to-exhaustion following supplementation. However, although it is a well-accepted measure of AC, MAOD does not allow for the isolated estimation of ePCr and has poor reliability (i.e., high limits of agreement). This may have hindered the detection of small differences in the anaerobic metabolism (Doherty et al., 2000, 2002).

Some studies (Bertuzzi et al., 2010; Zagatto et al., 2016; Miyagi et al., 2017) have proposed an alternative method to estimate the AC, denominated alternative MAOD. This method determines the AC through the sum of energetic equivalents of the net blood lactate accumulated during exercise and the fast component of excess post-exercise oxygen consumption (EPOC_fast_), which allows estimation of the contribution from the glycolytic (e[La^-^]) and ePCr pathways, respectively. However, the alternative MAOD does not measure oxygen deficit, thus we will call it AC estimated by the non-oxidative energetics sum measured during a single supramaximal effort (AC_[La-]+EPOCfast_). Some studies have demonstrated that AC_[La-]+EPOCfast_ is not different and is significantly correlated with conventional MAOD determined during running and cycling (Bertuzzi et al., 2010; Zagatto et al., 2016, 2017b; Miyagi et al., 2017), furthermore, AC_[La-]+EPOCfast_ is reliable and sensitive to discriminate individuals with distinct training levels (Zagatto et al., 2017b). Previous studies have also shown that AC_[La-]+EPOCfast_ can be sensitive to detect small changes in the anaerobic metabolism with intake of ergogenic supplements, such as those brought about by caffeine and sodium bicarbonate ingestion (Brisola et al., 2015; de Poli et al., 2016). Together, these findings suggest that AC_[La-]+EPOCfast_ could be more sensitive to detect small changes when compared to conventional MAOD.

Therefore, the study aimed investigate the effects of short-duration creatine monohydrate supplementation on AC measured by AC_[La-]+EPOCfast_ and MAOD, and on anaerobic energy pathways (i.e., ePCr and e[La^-^]), and time-to-exhaustion during high-intensity running. Since it is well documented that creatine supplementation can increase intramuscular phosphorylcreatine, we hypothesized it could increase the contribution of the phosphagen metabolism (i.e., ePCr) during high-intensity exercise, therefore improving performance and AC. This study makes progress in the current literature by investigating the effect of creatine supplementation through a novel method to estimate the AC and, particularly, the effects on non-mitochondrial pathway estimation, which has been hypothesized but not scientifically reported until the current date.

## Materials and Methods

### Participants

Eighteen male volunteers were initially enrolled in the study; however, four were excluded, thus, fourteen men [mean ± SD; age 24 ± 4 years; height 173.8 ± 6.2 cm; total body mass 73.4 ± 7.4 kg] completed participation in the study. To be included, volunteers were required to be recreationally active, participate in exercise activities such as running, cycling, and soccer at least 2 times per week, and not have used ergogenic supplements such as beta-alanine and creatine, among others, for at least 6 months. Volunteers who were regularly absent from the trials or presented injuries were excluded from the study.

The volunteers were instructed not to ingest alcohol, caffeine, and sodium bicarbonate and not to perform strenuous exercise 24 h before each trial. Volunteers were also verbally informed about the experimental procedures and signed an informed consent prior to beginning the study. All experimental procedures were approved by the Human Research Ethics Committee from the School of Sciences, São Paulo State University – UNESP (protocol number: 61323916.5.0000.5398) and the study was conducted in accordance with the Declaration of Helsinki.

### Experimental Design

**FIGURE 1:**
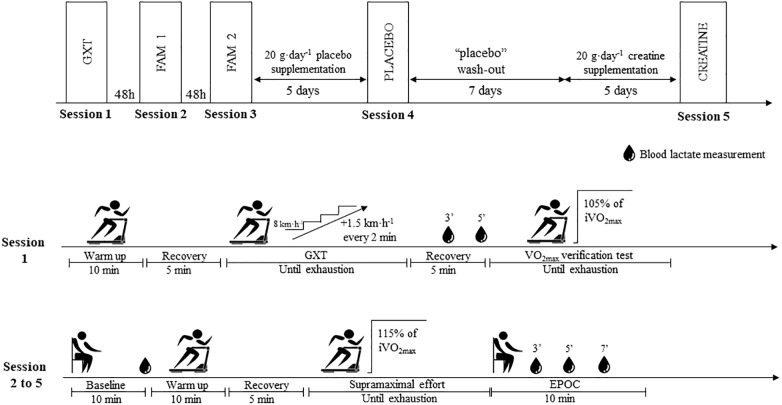
Experimental design of study. GXT, Graded exercise test; FAM, Familiarization; EPOC, Excess post-exercise oxygen consumption.

The investigation was conducted in a single-blind, placebo-controlled, crossover trial. Tests were performed on a motorized treadmill (ATL, Inbramed, Porto Alegre, Brazil) with a fixed gradient of 1% (Jones and Doust, 1996), using a safety belt to avoid accidental falls and to allow for maximal effort. In addition, the treadmill had been previously calibrated according to Padulo et al. (2014). Firstly, the volunteers performed a graded exercise test (GXT) to assess $\dot{V}$O_2max_ and the lowest running speed associated with $\dot{V}$O_2max_ (i$\dot{V}$O_2max_), followed by four sessions on different days of supramaximal constant workload tests at 115% of i$\dot{V}$O_2max_ to assess AC_[La-]+EPOCfast_ and oxygen uptake ($\dot{V}$O_2_) integral area (Zagatto et al., 2016; Miyagi et al., 2017). The first two supramaximal sessions served as familiarization trials. The third supramaximal test was performed under the placebo condition and the fourth test under the creatine condition (12 days after the placebo test). We opted to use the single-blind design with treatment order not being counterbalanced due to the long wash-out period necessary for muscle creatine to return to pre-supplementation values (Hultman et al., 1996). The experimental design of the study is presented in Figure 1.

In addition, 5 submaximal efforts were performed as warm-ups and used to construct the linear regression to allow determination of MAOD. All exhaustive sessions were separated by a minimum interval of 48 h. All participants were verbally encouraged to perform their maximal efforts in all sessions, and all tests were performed at the same time of day to avoid circadian variations in performance and AC (Hill, 2014).

### Physiological and Metabolic Measurements

Respiratory variables were measured breath-by-breath using a gas analyzer (Quark PFT, COSMED, Rome, Italy) calibrated following the manufacturer’s instructions. Heart rate was monitored using a transmission belt connected to the gas analyzer (Wireless HR 138 Monitor, COSMED, Rome, Italy). The respiratory variables and heart rate variables were smoothed every 5 points and interpolated second by second to reduce “noise” and increase the reliability of the parameter estimation, as previously reported for assessing MAOD and AC_[La-]+EPOCfast_ (Zagatto et al., 2016; Miyagi et al., 2017).

Blood samples were collected from the earlobe (25 μL) at rest (i.e., before the supramaximal effort) to measure the baseline blood lactate concentration ([La-]_BL_), 3 and 5 min after the GXT, and 3, 5, and 7 min after the supramaximal tests to determine the peak blood lactate concentration ([La-]_peak_). Blood samples were stored at –20°C in tubes containing 50 μL of 1% sodium fluoride, being subsequently analyzed in duplicate [Standard Error 0.27 mmol⋅L^-1^; Δ% -3.84 (CI95% -2.96 to -5.33)] using an automated analyzer (Yellow Springs Instruments model 2900, OH, United States).

### Graded Exercise Test (GXT) and Maximal Oxygen Uptake ($\dot{V}$O_2max_) Determination

The GXT was designed to last ∼8–12 min, according to guidelines to assess the $\dot{V}$O_2max_ and i$\dot{V}$O_2max_ (Howley et al., 1995). The GXT started at 8 km⋅h^-1^, with 1.5 km⋅h^-1^ increments every 2 min until exhaustion (Brisola et al., 2015; de Poli et al., 2016; Zagatto et al., 2016). Exhaustion was defined as the incapacity to maintain exercise intensity. After GXT, the participants remained for 5 min in passive recovery, and then returned to the treadmill to run until exhaustion at a workload corresponding to 105% of the maximal intensity reached in the GXT, which was used as verification testing to confirm $\dot{V}$O_2max_ (Rossiter et al., 2006).

The $\dot{V}$O_2_ average of the final 30 s in each GXT stage and 15 s in the rectangular test was calculated. $\dot{V}$O_2max_ was assumed when the $\dot{V}$O_2_ plateau was observed (difference of ≤2.1 mL⋅kg⋅min^-1^ between the last two complete stages of GXT). When no plateau could be observed, the highest average of $\dot{V}$O_2_ obtained during the GXT was compared with $\dot{V}$O_2_ reached in the rectangular test; $\dot{V}$O_2max_ being assumed as the highest average of $\dot{V}$O_2_ when not different from the $\dot{V}$O_2_ reached in the rectangular test (Rossiter et al., 2006). The minimal exercise intensity at which the subject reached $\dot{V}$O_2max_ was considered as i$\dot{V}$O_2max_.

### Supplementation Protocol

Twenty-four hours after the final familiarization test, the volunteers received 20 g⋅day^-1^ of placebo (dextrose) for 5 days for the “placebo” test session, followed by a 7 day “fake” washout period. Subsequently, creatine monohydrate (Creapure^®^, AlzChem AG, Germany, 20 g⋅day^-1^) was administered for another 5 days for the “creatine” test session. During supplementation and the washout period, the volunteers maintained their recreational exercise routine.

Supplements were given in 4 equal doses and the volunteers were instructed to ingest the supplements immediately after their main meal of the day. The placebo was given before creatine in a single blind design (i.e., only the participants were blinded) to avoid any carry over effect of creatine, considering its long-term washout period. The dose of creatine was chosen according to Harris et al. (1992) and Jacobs et al. (1997), who demonstrated that this dosage was sufficient to saturate the intramuscular creatine stores, and increase AC and time-to-exhaustion (tlim) in a supramaximal rectangular effort, respectively. Placebo and creatine supplements were identical in appearance, and were administered in flavored tablets containing 1 g of creatine and 2 g of dextrose each. At the end of the study, to test the efficacy of the blinding design, volunteers were asked about which arm of the study they had received the creatine supplement in. Only 5 out of the 14 volunteers correctly answered when they ingested creatine.

### Submaximal and Supramaximal Efforts

Baseline $\dot{V}$O_2_ was measured with volunteers seated for 10 min before the tests. Subsequently, the volunteers performed five 10-min submaximal efforts at 30, 40, 50, 60, and 70% of i$\dot{V}$O_2max_, with the first three intensities performed as warm-up, 5 min before the supramaximal tests. The submaximal efforts at 60 and 70% of i$\dot{V}$O_2max_ were performed on different days, so as not to interfere with the performance of the supramaximal effort.

Supramaximal efforts at 115% were performed until voluntary exhaustion or the inability to continue the exercise, and the tlim was recorded. The choice of intensity of the supramaximal test (i.e., 115%) was based on previous investigations which demonstrated that this intensity is the highest exercise intensity to determine the AC_[La-]+EPOCfast_ (Zagatto et al., 2016; Miyagi et al., 2017, 2018). Immediately after the end of the supramaximal tests, the participants remained seated quietly for 10 min for measurement of EPOC_fast_. The supramaximal efforts were performed 4 times, the first 2 efforts being used as familiarization and the next 2 efforts after the placebo and creatine ingestion periods. The final familiarization supramaximal test was compared with the placebo condition to ensure that there was no longer any familiarization effect.

### Conventional MAOD Determination

A linear regression was fitted using 5 submaximal intensities and respective $\dot{V}$O_2_, considering the $\dot{V}$O_2_ average 8–10 min, with the y-intercept fixed at the baseline $\dot{V}$O_2_ (Özyener et al., 2003). This linear regression was extrapolated to estimate the oxygen demand at 115% i$\dot{V}$O_2max_ (Zagatto et al., 2016; Miyagi et al., 2017). In addition, the area of $\dot{V}$O_2_ measured during the supramaximal intensity was determined using the trapezoidal method (Medbø et al., 1988; Zagatto et al., 2016; Miyagi et al., 2017). Therefore, the MAOD was considered as the difference between the estimated oxygen demand area (estimated oxygen demand at 115% of i$\dot{V}$O_2max_ multiplied by tlim) and the area $\dot{V}$O_2_ of the supramaximal effort (Medbø et al., 1988).

### AC_[La-]+EPOCfast_ and Non-mitochondrial Pathways Measurements

The AC_[La-]+EPOCfast_ was assumed as the sum of ePCr and e[La^-^] estimated during the supramaximal effort (Bertuzzi et al., 2010; Zagatto et al., 2016, 2017a; Miyagi et al., 2017; Redkva et al., 2018). The ePCr was estimated by the product between $\dot{V}$O_2_ amplitude (A1) and time constant (τ1) from a first exponential decay fitted using a bi-exponential fit in EPOC_fast_, with OriginPro 8.0 software (Origin Lab Corporation, Microcal, MA, United States) (Bertuzzi et al., 2010; Zagatto et al., 2016, 2017a; Miyagi et al., 2017), while e[La^-^] was estimated using the net lactate accumulation (i.e., difference between [La-]_peak_ and [La-]_BL_), considering for each 1 mmol⋅L^-1^ of the net blood lactate an equivalent of 3 mL⋅kg^-1^ of oxygen (Di Prampero and Ferretti, 1999). The oxidative pathway (eOXID) was assumed as the $\dot{V}$O_2_ integral under the curve (i.e., $\dot{V}$O_2_ area) subtracting the baseline $\dot{V}$O_2_ area.

### Statistical Analyses

The data are presented as mean ± standard deviation and 95% confidence interval (CI95%). All variables were examined using the Shapiro–Wilk test to check for normal distribution. To determine tlim reliability after familiarization, the intraclass correlation coefficients were applied (Koo and Li, 2016). The paired “t” test was used to compare the values of MAOD and AC_[La-]+EPOCfast_ for each condition. Pearson’s correlation test was also applied to determine the associations between values of MAOD and AC_[La-]+EPOCfast_ for each condition. The Pearson’s correlation test was applied and the coefficient of correlation was classified as negligible (0 to 0.2), weak (≥0.2 to 0.4), moderate (≥0.4 to 0.7), strong (≥0.7 to 0.9), and very strong (≥0.9 to 1.0) (Rowntree, 1991). In all cases, a significance level of 5% was assumed. As a qualitative analysis, the magnitude of differences between groups was calculated and expressed as standardized mean differences (Cohen’s d), assuming threshold values for Cohen’s d statistics of ≥0.2 (small), ≥0.5 (moderate), and ≥0.8 (large) (Cohen, 1988). In all cases, a significance level of 5% was assumed. The smallest worthwhile change was calculated as the product between the standard deviation between subjects in the placebo condition and 0.2, to verify the change in substantial or harmful effect of creatine.

## Results

Peak and maximal variables measured during the GXT and verification testing are shown in Table 1. The peak $\dot{V}$O_2_ measured during the GXT and during the verification testing at 105% to confirm the $\dot{V}$O_2max_ was not different (P = 0.19), confirming that $\dot{V}$O_2max_ was measured.

**Table 1:** GXT and rectangular exercise test parameters.

Variables	
Exercise Duration (min)	11.20 ± 1.52 (10.33 to 12.08)
Rectangular exercise duration (min)	2.20 ± 0.33 (2.01 to 2.39)
$\dot{V}$O_2max_ reached in GXT (mL⋅kg^-1^⋅min^-1^)	49.25 ± 3.74 (47.09 to 51.41)
$\dot{V}$O_2max_ reached in rectangular test (mL⋅kg^-1^⋅min^-1^)	48.76 ± 4.18 (46.34 to 51.17)
i$\dot{V}$O_2max_ (km⋅h^-1^)	14.9 ± 1.2 (14.2 to 15.5)
Respiratory exchange ratio	1.15 ± 0.05 (1.12 to 1.17)
[La^-^]_PEAK_ (mmol⋅L^-1^)	9.20 ± 2.09 (7.99 to 10.41)


*Values are mean ± SD (CI95%)*.

Considering the values of i$\dot{V}$O_2max_ achieved in the GXT, the velocity corresponding to 115% applied during the supramaximal tests was 17.1 ± 1.3 (CI95% = 16.3 to 17.9) km⋅h^-1^. The tlim in the final familiarization supramaximal test was compared with the placebo condition, and demonstrated strong reliability (ICC = 0.86; P < 0.001).

**FIGURE 2:**
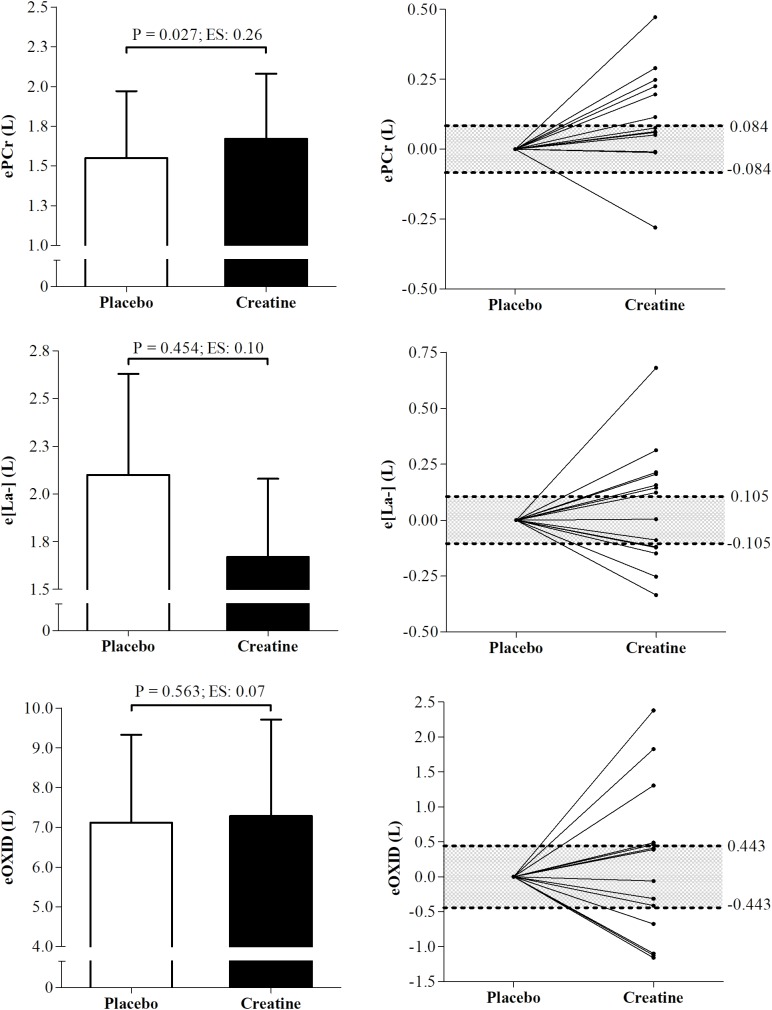
Differences and individual smallest worthwhile change of energetics data from phosphagen, glycolytic, and oxidative pathways under placebo and creatine conditions. ePCr, energetics from the phosphagen systems; e[La^-^], energetics from glycolytic pathway; eOXID, oxidative phosphorylation pathway; ES, effect size.

The ePCr increased significantly in the creatine condition when expressed in absolute values (P = 0.027; ES = 0.26), besides which, 7 participants were responsive to creatine supplementation according to the smallest worthwhile change analysis. However, e[La^-^] and eOXID were not altered (P = 0.45; ES = 0.10 and P = 0.56; ES = 0.07, respectively) even though 7 participants were responsive to creatine supplementation for these variables (Figure 2).

In addition, there were no differences between creatine and placebo conditions in tlim and in the ePCr, e[La^-^], and eOXID when expressed in percentages of total energetics contribution (Table 2).

**Table 2:** Performance and percentage of metabolic energetics during the supramaximal effort in placebo and creatine conditions.

	Placebo	Creatine	Δ%	P
tlim(s)	160.79 ± 37.76	163.64 ± 38.72	+2.27	0.49
	(138.98 to 182.59)	(141.29 to 186.00)		
%e[La^-^]	19.89 ± 3.17	19.82 ± 3.83	–0.06	0.91
	(18.06 to 21.72)	(17.61 to 22.03)		
%ePCr	15.09 ± 4.69	15.74 ± 4.04	+0.65	0.25
	(12.38 to 17.79)	(13.40 to 18.07)		
%eOXID	65.02 ± 6.20	64.44 ± 6.48	–0.59	0.49
	(61.44 to 68.60)	(60.69 to 68.18)		


*Values of creatine and placebo condition are expressed as Mean ± SD (95%IC). tlim, time to exhaustion; %e[La^-^], percentage of energetics from glycolytic pathway; %ePCr, percentage of energetics from the phosphagen systems; %eOXID, percentage of energetics from oxidative phosphorylation pathway*.

**FIGURE 3:**
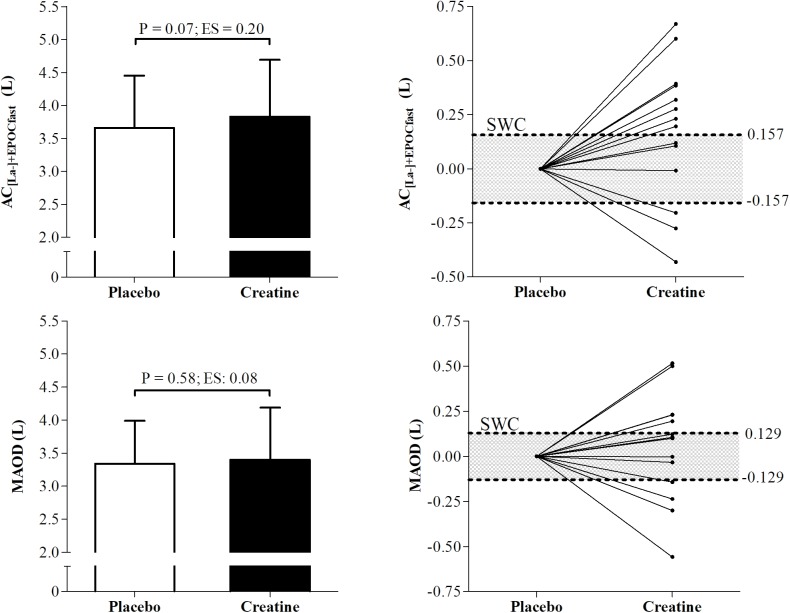
Differences and individual smallest worthwhile change of anaerobic capacity (AC) measured by AC_[La-]+EPOCfast_ and MAOD. AC_[La-]+EPOCfast_, AC estimated by non-oxidative energetics sum measured during a single supramaximal effort; MAOD, maximal accumulated oxygen deficit; ES, Effect size; SWC, Smallest worthwhile change.

Figure 3 presents means (±SD) and individual smallest worthwhile change values of AC measured using MAOD and AC_[La-]+EPOCfast_ methods under creatine and placebo conditions. The AC measured by MAOD and AC_[La-]+EPOCfast_ did not present significant differences between placebo and creatine conditions (P = 0.58 and P = 0.07). However, in the effect size (ES), the AC_[La-]+EPOCfast_ showed a small positive effect of creatine supplementation on AC (ES = 0.20; Δ% = 4.90%), while the MAOD presented a negligible effect size (ES = 0.08; Δ% = 1.19%). Furthermore, 8 participants were positive responders to creatine supplementation for AC_[La-]+EPOCfast_ and 5 for MAOD.

**FIGURE 4:**
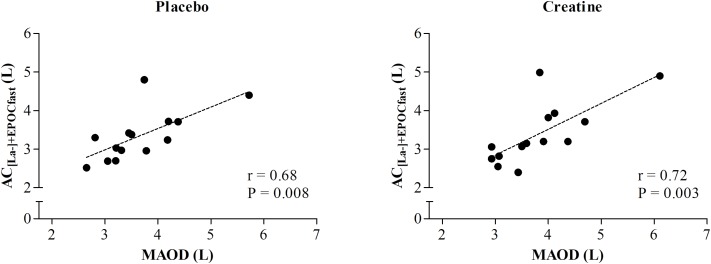
Correlation between AC estimated by non-oxidative energetics sum measured during a single supramaximal effort (AC_[La-]+EPOCfast_) and through MAOD.

In addition, MAOD and AC_[La-]+EPOCfast_ were not different (P = 0.08) under the placebo condition and showed a moderate and significant correlation (r = 0.68; P = 0.008). However, under the creatine condition, these variables also presented a significant and strong correlation (r = 0.72; P = 0.003), although the AC measured using the AC_[La-]+EPOCfast_ method was greater than the conventional MAOD procedure (P = 0.02) (Figure 4).

When asked which supplement they took at each moment, only 5 out of the 14 volunteers correctly answered when they had ingested creatine, thus demonstrating that blinding of the study was effective for the majority of volunteers.

## Discussion

In the present study we investigated the effects of short-duration creatine monohydrate supplementation on AC measured using AC_[La-]+EPOCfast_ and conventional MAOD methods. The main findings of the present study were the increase in ePCr and consequent small effect on AC measured by AC_[La-]+EPOCfast,_ with 8 of 14 positive responders, despite the non-significant finding (P = 0.07) Despite this, performance in the supramaximal effort at 115% of i$\dot{V}$O_2max_ and AC measured by MAOD were not changed.

It is already known that monohydrated creatine short-duration supplementation may increase creatine intramuscular stores by ∼20%, the phosphorylcreatine resynthesis rate, and ATP availability for the phosphagen pathway (Harris et al., 1992; Greenhaff et al., 1994; Terjung et al., 2000), thus being a possible mechanism to explain the increase in ePCr verified in the present study. Although we did not measure creatine content in muscle and ATP availability, the rate of increase in ePCr corroborates previous findings, since even though the intramuscular content of creatine may increase at a higher rate (i.e., 10–20%) after creatine administration, the energy supply increases at a lower rate (i.e., ranging from 2.5 to 10%), being dependent on the intensity and duration of the effort (Terjung et al., 2000).

Despite the non-significant effect of creatine supplementation on AC measured by AC_[La-]+EPOCfast_ and MAOD, a slight positive effect of creatine was verified only in AC_[La-]+EPOCfast_ (ES = 0.20), indicating the responsiveness of this method, since the majority of participants were responsive to creatine administration (8 of 14 participants). Although non-significant, the slight increase could be directly attributed to the increase in ePCr, as the ePCr corresponds to approximately 40% of AC_[La-]+EPOCfast_ in moderately active individuals (Zagatto et al., 2017b). Jacobs et al. (1997) reported improvements in AC measured by the conventional MAOD method and in performance at 125% of i$\dot{V}$O_2max_ intensity, after 5 days of creatine supplementation (20 g⋅day^-1^), not supporting our results (i.e., no differences in MAOD or tlim). However, Doherty et al. (2002), using the same intensity, did not find differences for the MAOD or tlim, corroborating with the results of the present study.

Although MAOD is considered the most accepted method for AC measurement (Medbø and Tabata, 1993; Noordhof et al., 2010), a possible explanation for the discordant results could be supported by the high values of limits of agreement (15.1 mL⋅kg^-1^) and poor reliability of the conventional MAOD method, making a larger sample size necessary to detect small differences (Doherty et al., 2000). On the other hand, AC_[La-]+EPOCfast_ presents lower limits of agreement (2.9 mL⋅kg^-1^) and coefficient of variation (4.1%) values (Zagatto et al., 2016; Miyagi et al., 2017), thus possibly explaining why AC_[La-]+EPOCfast_, although with no significant change (P = 0.07), was able to detect a small positive impact of creatine supplementation compared with the placebo condition (ES = 0.20), differing from the MAOD result (P = 0.58; ES = 0.08). In addition, recently Brisola et al. (2015) and de Poli et al. (2016) verified improvement in AC_[La-]+EPOCfast_ caused by acute sodium bicarbonate and caffeine supplementation, suggesting the “efficiency” of the method to detect slight changes caused by ergogenic sources. However, it should be mentioned that the possibly higher sensitivity of AC_[La-]+EPOCfast_ when compared with MAOD has to be assumed carefully, since our results present a small effect on this variable. Thus, further studies are necessary to elucidate the sensitivity of AC_[La-]+EPOCfast_ and MAOD to detect changes caused by creatine intake compared with a direct method (i.e., muscle biopsy). In addition, both the placebo and creatine conditions showed a significant and moderate correlation between AC_[La-]+EPOCfast_ and MAOD (r = 0.68 and 0.72, respectively), corroborating with the results of Zagatto et al. (2016) (r = 0.73) and Miyagi et al. (2017) (r = 0.68). It should also be mentioned that the significant difference between AC_[La-]+EPOCfast_ and MAOD under the creatine condition (P = 0.02) added to the maintenance of a moderate correlation between the methods.

Despite the increase in ePCr and the small effect on AC measured by AC_[La-]+EPOCfast_ after monohydrated creatine supplementation, performance in the supramaximal effort was not changed. This result is also divergent from those reported by Jacobs et al. (1997), which could be largely attributed to the supramaximal intensity, since we used 115% of i$\dot{V}$O_2max_ (i.e., scientifically backed assessment for AC_[La-]+EPOCfast_ determination) (Zagatto et al., 2016; Miyagi et al., 2017), and Jacobs et al. (1997) used 125% of i$\dot{V}$O_2max_, which could increase the portion of the energy supply by ePCr during the effort and its importance for the performance (Terjung et al., 2000; Mesa et al., 2002), as the present study showed that ePCr supplies a small portion of the total energy during the 115% of i$\dot{V}$O_2max_ effort (Table 2).

In addition, the values of e[La^-^] were not altered after creatine supplementation. This result was expected as there is no evidence that creatine supplementation increases the glycogen content or glycolysis activity, corroborating the results of Doherty et al. (2002), although Zuniga et al. (2012) reported an increase in ATP production through the non-oxidative glycolytic pathway in the Wingate anaerobic test after creatine supplementation, attributing this result to the creatine buffering effect. The eOXID also remained unchanged, probably due to the lack of improvement in performance.

The main limitation of the present study was the lack of randomization of the tests. However, it is noteworthy that although no randomization was performed, two familiarizations were carried out before starting all procedures to minimize a possible “learning effect,” and during the test, no difference was verified between the last familiarization and the placebo condition. Therefore, despite this being a study limitation, the lack of randomization seems not to have affected our findings.

## Conclusion

In summary, short-term monohydrated creatine supplementation (20 g⋅day^-1^ for 5 days) improves the ePCr, although it is not enough to significantly improve the AC measured by AC_[La-]+EPOCfast_ and MAOD, or performance during a supramaximal effort.

## Author Contributions

RdP collected and analyzed the data, and wrote the manuscript. LR collected the data. EM wrote the manuscript. GA, RB, and AZ conceived the idea, built the experimental design, and wrote the manuscript.

## Conflict of Interest Statement

The authors declare that the research was conducted in the absence of any commercial or financial relationships that could be construed as a potential conflict of interest.
